# Hybrid Pectin-Based Sorbents for Cesium Ion Removal

**DOI:** 10.3390/ma13092160

**Published:** 2020-05-07

**Authors:** Joanna Bok-Badura, Agata Jakóbik-Kolon, Alicja Kazek-Kęsik, Krzysztof Karoń

**Affiliations:** Faculty of Chemistry, Silesian University of Technology, Krzywoustego 6, 44-100 Gliwice, Poland; agata.jakobik@polsl.pl (A.J.-K.); alicja.kazek-kesik@polsl.pl (A.K.-K.); krzysztof.karon@polsl.pl (K.K.)

**Keywords:** pectin, sorption, cesium ion removal, Prussian blue, pectin-based biosorbent

## Abstract

In this paper, beads-shaped hybrid sorbents composed of pectin and Prussian blue were prepared. Various ratios of pectin and Prussian blue in hybrid sorbents were tested. Obtained sorbents had high and roughly constant sorption capacity in a broad pH range (4–10), in which also the swelling index and stability of sorbents were satisfactory. The preliminary sorption studies proved that almost 100% of cesium removal efficiency may be achieved by using the proper sorbent dose. The sorption capacity of the hybrid sorbent with a 1:1 ratio of pectin to Prussian blue equaled q = 36.5 ± 0.8 mg/g (dose 3 g/L, pH = 6, temp. = 22 ± 1 °C, t = 24 h). The obtained results showed that the prepared hybrid pectin-based sorbents are promising for cesium ions removal.

## 1. Introduction

Cesium-137, due to its long half-life of 30.17 years and radioactivity, is a serious threat to health. Due to its high solubility, high mobility, and easy incorporation into living organisms, cesium may cause various human diseases (e.g., thyroid cancer and birth defects) [1,2,3]. It is present in spent nuclear fuel and may be released to the environment due to the failure of nuclear power plants. Cesium may also come from the production and testing of nuclear weapons or the production and use of radioisotopes in medicine and industry [4,5]. Therefore, methods and materials for cesium ion removal are required. 

To date, methods such as chemical precipitation, extraction, volatilization, adsorption, and ion exchange have been proposed for cesium removal [6,7]. Both liquid-liquid solvent extraction and precipitation usually generate large quantities of secondary radioactive waste. Adsorption and ion exchange, both seem to be the most simple, economical, and effective techniques, especially if the concentration of metal ions in solution is low (below 100 mg/L). A number of sorbents have been proposed to date [8,9,10,11,12,13,14,15,16,17]; however, the most widely known materials for selective cesium removal are the metal hexacyanoferrates and their composites. Ferric hexacyanoferrate (Prussian Blue, PB) was successfully used after the Chernobyl nuclear reactor disaster [16]. Despite the high selectivity of Prussian blue and its analogues towards cesium ions, their use can be difficult. This is mainly due to their form, i.e., fine powders. There are problems with the separation of the sorbent from the purified solution and the use of a dynamic column system. Fine particles may form a dense cake, which is difficult to filter, and makes it almost impossible to pump the solution through a packed column. The usage of metal hexacyanoferrates in granular form may also be impractical due to factors such as their irregular shape or poor mechanical stability [18]. These problems may be overcome by the application of composites in which the active material (metal hexacyanoferrate) is encapsulated into a polymer matrix or sited on a support. For example, Dwivedi et al. prepared potassium cobalt hexacyanoferrate-gel beads in which sodium alginate containing PVA was used as a binding matrix. The sorption capacity of the obtained material was 15 mg/g [18]. Similar studies were conducted by Vipin et al. They used sodium cobalt hexacyanoferrate and carbon nanotubes encapsulated in alginate vesicles for cesium and strontium removal [19]. Calcium alginate was also used for immobilization of PB/Fe_3_O_4_/GO nanocomposites. Obtained microbeads were of 2.9 to 3.1 mm in diameter and of 43.52 mg/g sorption capacity towards cesium ions [20]. Nilchi et al. proposed a copper hexacyanoferrate-polyacrylonitrile composite for cesium removal; this sorbent had an adsorption capacity of 25.5 mg/g [21]. Unfortunately, some of the mentioned sorbents require the use of a lot of chemicals, and the synthesis procedures are multistage and complicated. This means that their mass production may be difficult and not cost-effective. Therefore, new selective and easily produced sorbents, especially in the form of beads, with good mechanical and operational parameters are still demanded. 

In this study, a hybrid inorganic-biopolymeric sorbents composed of Prussian blue and cross-linked pectin for cesium ions removal were proposed.

Pectin is a natural polysaccharide obtained mainly as a byproduct from apple or citrus peels. It is readily accessible, cheap, nontoxic, and ecofriendly. The chemistry of pectin is known and has been much discussed [22,23]. The primary pectin polymer chain is mainly built of galacturonic acid units joined by (1,4)-α bonds. The unique structure of pectin and its gelling property (e.g., in the presence of calcium ions) allows insoluble, interpenetrating pectin gels to be obtained. The mechanism of pectin gelation by calcium ions is commonly described by the egg-box model, which perfectly describes the gelation of other polysaccharide-alginates. Although the gelation result of these polysaccharides in the presence of calcium ions is the same, the mechanism of gelation of pectin is slightly different from that of alginate which was presented by Braccini and Perez [24]. Nevertheless, the pectin gels shown high stability. Pectin biosorbents were used for removal of various heavy metals, e.g., Pb(II), Cd(II), and Cu(II), from water and wastewater [25,26]. It was also proved that pectin may be a good matrix material for the immobilization of other sorption substances, e.g., titanium dioxide or modified poly(methyl methacrylate) waste [27,28]. There is no information concerning immobilization of Prussian blue in pectin matrix to create sorption beads. Pectin was only proposed as a stabilizer in pectin-stabilized magnetic graphene oxide Prussian blue nanocomposite. The function of pectin in this material was improving the graphene oxide sheets separation and enhancing their dispersion. The final material was obtained by being ground to a uniform size, into the form of a powder [29]. We believe that a combination of sorption and gelation properties of pectin and sorption properties of PB allow the obtainment of a unique hybrid pectin-based sorbent for cesium ion removal.

## 2. Materials and Methods 

### 2.1. Materials and Reagents

For Prussian blue (PB) preparation, FeCl_3_ 6H_2_O (Sigma-Aldrich, Darmstadt, Germany) and K_4_[Fe(CN)_6_] 10H_2_O (Sigma-Aldrich) were used. A commercial PB powder (Alfa Aesar, ThermoFisher GmbH, Kandel, Germany) was used for comparison. Cesium nitrate (Sigma-Aldrich) was used for Cs solution preparation. Amide pectin (type NECJ A2, esterification degree 30.2%, amidation degree 19.9%) (C&G Spółka z o.o., Jasło, Poland) and calcium chloride (Chempur, Piekary Śląskie, Poland) were used for beads preparation. Nitric acid (“Suprapur” from Merck, Darmstadt, Germany) and sodium hydroxide (Avantor, Gliwice, Poland) were used for pH adjustment. A certified reference solution of Cs in HNO_3_ (1000 ± 3 μg/mL) from Peak Performance, USA, was used for calibration. The water used for the experiments was purified using a Millipore Elix 10 (Milipore SAS, Molsheim, France) system.

### 2.2. Apparatus

Depending on cesium ions concentration, their determination was performed using a Varian 710-ES inductively coupled plasma atomic emission spectrometer (ICP-AES) (Varian, Palo Alto, CA, USA) or a Varian AA240FS atomic absorption spectrometer (AAS) (Varian) equipped with a single-element Atomax™ hollow cathode lamp (HCL) for the detection of elemental Cesium (Cs) (PerkinElmer, Waltham, MA, USA). The parameters for cesium analysis using the ICP-AES apparatus were as follows: RF power 1.0 kW, plasma flow 15 L/min, auxiliary flow 1.5 L/min, nebulizer pressure 200 kPa, pump rate 15 rpm, and emission lines λ_Cs_ = 672.328 and 697.327 nm. The parameters for iron analysis using the ICP-AES apparatus were as follows: RF power 1.0 kW, plasma flow 15 L/min, auxiliary flow 1.5 L/min, nebulizer pressure 200 kPa, pump rate 15 rpm, and emission lines λ_Fe_ = 238.204 and 259.940 nm. The parameters for cesium analysis using the AAS apparatus were as follows: air/acetylene, wavelengths 852.1 and 455.5 nm, and slit widths 1.0 and 0.5 nm, respectively. For the preparation of Prussian blue powders, hybrid sorbents, and for sorption studies a thermostatted shaker (Incu-Shaker, Benchmark, Sayreville, NJ, USA), a peristaltic pump (Catalyst FH100M 8/3, Cole-Parmer North America, Vernon Hills, IL 60061, USA), a Tube Mill 100 control batch mill with disposable grinding chambers (IKA Werke, Staufen im Breisgau, Germany), and an MPV-350 centrifuge (MPW MED. INSTRUMENTS, Warsaw, Poland) were used.

### 2.3. Sorbent Preparation and Charactrization 

#### 2.3.1. Prussian Blue Preparation 

Prussian blue can be prepared by the reaction of K_4_[Fe(CN)_6_] and FeCl_3_. In this study PB was prepared from 10% aqueous solutions of K_4_[Fe(CN)_6_] and FeCl_3_ in various molar ratios (1:1.15, 1:1.5, and 1:2.3). The solutions were stirred for at least 1 h at room temperature, and the obtained slurries were then centrifuged (9000 rpm, 5 min), decanted, and the precipitates were washed with deionized water (eight times). Finally, the obtained precipitates were dried at 40, 80, or 105 °C, and ground using a batch mill and mortar. Six PB powders, marked as O, O1, O2, O3, O4, O5, O6 were obtained.

#### 2.3.2. Hybrid Sorbents’ Preparation

To obtain the sorbents with a c.a. of 10%, 30%, or 50% (m/m) content of PB, an adequate amount of the obtained PB powder was dispersed in water, and the resultant slurry was rapidly mixed with the correct amount of pectin solution (the concentration of pectin in the slurry was 3% (m/m)). The obtained slurry was then slowly added by a peristaltic pump into a continuously stirred, cold (ca. 4 °C) 1M CaCl_2_ solution to form round, dark blue beads. The obtained material was left in the mother solution at ca. 4 °C overnight, and then the beads were filtered and washed with deionized water several times to remove chloride ions. Finally, the beads were dried at 40 °C for 24 h. Three sorbents, with the established amount of PB at 10%, 30% and 50% were prepared and marked as S1, S2 and S3, respectively. Simultaneously, a pectin-only sorbent was prepared (S). 

#### 2.3.3. Materials Characterization

The obtained powders, as well as the hybrid sorbents and the pectin-only sorbent, were examined using thermogravimetric analysis (MOM Q-1500D, Paulik, Paulik and Erdey, MOM, Budapest, Hungary). The measurement was performed in air from ambient conditions to 1000 °C at a heating rate of 5 °C/min. The phase compositions of the ground hybrid sorbent, the pectin-only sorbent, and the PB powders were determined using powder X-ray diffraction (XRD) (Seifert 3003TT powder X-ray diffractometer, RICH. SEIFERT & CO. GmbH & Co. KG, Ahrensburg, Germany) with a copper X-ray tube (kλ1 = 1.540598 Å, kλ2 = 1.544426 Å, and kβ = 1.39225 Å; Ahrensburg, Germany). The XRD patterns were recorded over a 2θ range of 5° to 80°. The morphologies of the hybrid sorbents were observed using a scanning electron microscope (Phenom Pro Desktop SEM—Phenom-World B.V., Eindhoven, The Netherlands) equipped with an EDS detector. Prior to the SEM analysis hybrid sorbent samples were freeze-dried using an Alpha 1-2 LD plus lyophilizer (Martin Christ Gefriertrocknungsanlagen GmbH, Germany).

To determine the swelling index of sorbent, 30 mg of sorbent was introduced into the cesium ion solution (10 mL, 115 mg/L) at various pH values and left for 24 h at a temperature of 22 ± 1 °C. The sorbent was then carefully dried using a paper towel and immediately weighed. The effect of pH on the swelling index of the obtained material was examined in a pH range of 4–10. The swelling index was calculated using the formula shown in Equation (1):

(1) $$S=\frac{m_{s}}{m_{d}}$$

where:

m_s_—mass of the swollen sorbent [g],
m_d_—mass of the dry sorbent [g].

The sorbent stability was determined by means of examining the leaching of iron ions from the sorbent into solution during sorption studies over a prolonged period of time. The amount of iron leached from the sorbent into solution was determined by ICP-AES after 1, 4, and 7 days of Cs ion sorption at various pH values. The percentage of leached iron was calculated based on the results from the thermogravimetric analysis (TG) and ICP-AES analysis. The total amount of iron in the sorbent samples was calculated based on the TG profiles of the PB powder and hybrid sorbent, assuming that only the insoluble Prussian blue (Fe_4_[Fe(CN)_6_]_3_) was obtained and its only decomposition residue was iron oxide. The percentage of leached iron (%_Fe_) was calculated using the formula shown in Equation (2):

(2) $${\%}_{\mathrm{Fe}}=\frac{m_{L}\times 100\%}{m_{\mathrm{Fe}}}$$

where:

m_L_—the mass of iron leached from a known amount of sorbent into solution,
m_Fe_—the mass of iron in the hybrid sorbent (calculated from TG analysis).

### 2.4. Preliminary Sorption Studies

#### 2.4.1. General Sorption Examination

The correct amount of PB powder or dry sorbent was introduced into the appropriate volume of cesium ion solution (non-radioactive) (115 mg/L, pH = 6) and mixed using a shaker (22 ± 1 °C). A non-radioactive cesium was used for safety reasons, and because it exhibits the same chemical behavior as the radioactive one [30,31]. After 24 h the powder or sorbent was filtered, and the concentration of cesium ions in the remaining solution was measured using the ICP-AES or AAS apparatus. The amount of sorbed cesium was calculated as the difference between the amount of cesium in the initial solution and that in the solution after sorption. The sorption capacity (q) was computed using the formula shown in Equation (3).

(3) $$q=\frac{[c_{0}-c]V}{m}$$

where:

c_0_—the initial concentration of cesium ions in the solution [mg/L],
c—the final concentration of cesium ions in the solution [mg/L],
V—the volume of the solution [L],
m—the mass of the dry biosorbent [g].

Preliminary sorption studies were performed as described above and using the following conditions: mass of sorbent: m = 30 mg, volume of cesium ions solution: V = 10 mL, cesium ions concentration: c = 115 mg/L, pH = 6, temperature: 22 ± 1 °C, time: t = 24 h.

#### 2.4.2. The Effect of the pH on Sorption Properties

The effect of the pH on cesium ion sorption was tested according to *General sorption examination*, with details as follows: 30 mg of sorbent was shaken (22 ± 1 °C, 24 h) with 10 mL of cesium ion solution (115 mg/L) at various pH values (2–10).

#### 2.4.3. The Effect of the Sorbent Dose on Sorption Capacity

Various amounts of sorbent (5–150 mg) were introduced into 20 or 50 mL of Cs ion solution (115 mg/L, pH = 6) and left for 24 h. After this time, the sorbent was filtered, and the concentration of cesium in the filtrate was measured by means of ICP-AES. The sorption capacity (Equation (3)) and the removal efficiency, R, (Equation (4)) were than calculated.

(4) $$R=\frac{\left[c_{0}-c\right]}{c_{0}}\times 100\%$$

where:

c_0_—the initial concentration of cesium ions in the solution [mg/L],
c—the final concentration of cesium ions in the solution [mg/L].

## 3. Results

### 3.1. Sorbent Preparation and Charactrization

#### 3.1.1. Prussian Blue Preparation and Characterization

The method of PB preparation, in which potassium ferrocyanide solution is slowly added to iron (III) chloride solution, is commonly known [32]. In our studies, the effect of various parameters, such as the molar ratio of substrates, the contact time with the mother solution, and the temperature of drying, on the sorption capacity of the PB powders was examined. Six PB powders were obtained (O, O1, O2, O3, O5). The conditions of PB preparation and their sorption capacity are shown in Table 1. 

An excess of FeCl_3_ was applied to ensure the insoluble PB’s precipitation [33]. We started with a 1:1.15 molar ratio, but during the washing most of the precipitate dissolved. An increase in the amount of FeCl_3_ to molar ratios of 1:1.5 and 1:2.3 resulted in good precipitates in both cases, which also presented a similar sorption capacity. In further studies, PB prepared from K_4_[Fe(CN)_6_] and FeCl_3_ in a molar ratio of 1:1.5 was used. Additionally, the obtained results confirmed that the drying temperature affects the sorption capacity of PB powders. Drying at higher temperatures reduces the sorption capacity by 24% and 34% (relative to O1 powder) for O4 and O5 powder, respectively. An extension of the components’ mixing time did not improve the sorption properties of the powder. Therefore, for further studies PB powder obtained by mixing the reagents for 1 h and drying at 40 °C (O1) was used. 

Comparison of our materials with these from the literature is difficult due to at least two reasons: firstly, the cesium adsorption capacity of PB-only powders is rarely presented, probably due to the problems with the separation of the fine PB powder from purified solutions; secondly, the sorption capacity is strongly dependent on its determination conditions, e.g. the sorbent dose. The only sorption capacity data found in the literature for PB-only powders are as follows: commercial Prussian blue (12.5 and 15 mg/g) [15,34], PB powder from 960 mM FeCl_3_·6H_2_O and 720 mM Na_4_[Fe(CN)_6_] dried at 90 °C (158.47 mg/g) [35], and PB granules (80% PB and 20% binder, 241 mg/g) [36]. It should be noted that in the above-mentioned examples the conditions in which the sorption capacity was determined varied each time, thus any comparison is inappropriate. Therefore, we checked the sorption properties of the commercially available PB powder, applying the same conditions as for the PB powders prepared by us. The sorption capacity of the commercial powder was almost half that of the synthesized powders (23.2 ± 0.1 mg/g). The results proved our PB to be better for cesium sorption than the commercial one. 

#### 3.1.2. Hybrid Sorbents’ Preparation and Characterization 

**Figure 1:**
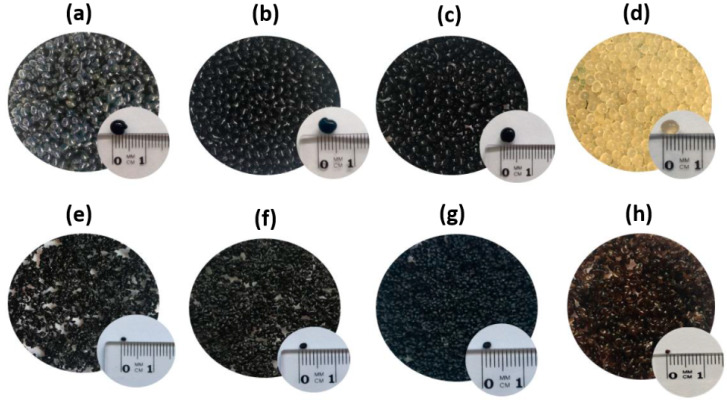
Pictures of prepared sorbents. Wet form: (**a**) S1, (**b**) S2, (**c**) S3, (**d**) S, and dry form: (**e**) S1, (**f**) S2, (**g**) S3, (**h**) S.

Sorbents containing c.a. 10% (S1), 30% (S2), and 50% (S3) of the prepared PB powder (O1) and the pectin-only sorbent (S) were obtained. The shape of all sorbents was the same-round, dark blue, homogenous beads (pectin-only beads were amber) of ca. 4 mm (wet) and 1–2 mm (dry) diameter (Figure 1). 

**Figure 2:**
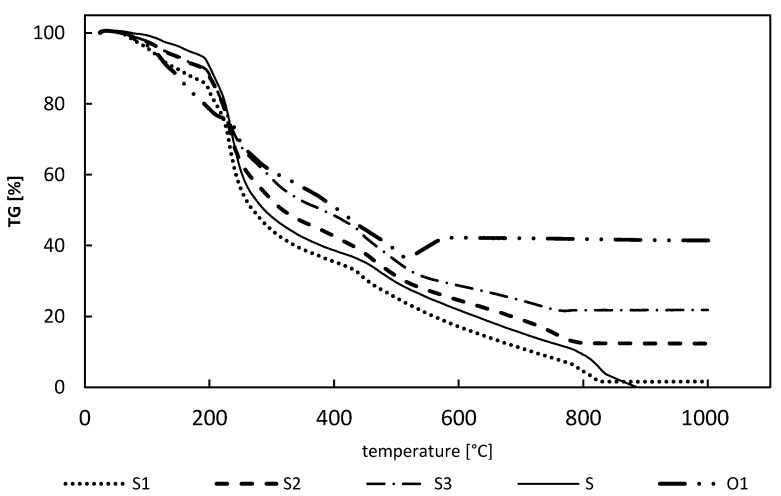
Thermogravimetric (TG) profiles of the hybrid gel beads (S1, S2, S3), pectin-only gel beads (S), and PB powder (O1).

For examination of the sorbents and PB powder (O1), TG analysis was used. The obtained TG profiles (Figure 2) proved the good thermal stability of the obtained hybrid sorbents up to 200 °C, as within this temperature range only water loss was observed. 

For all the sorbents, as well as the PB-only powder, the first region of mass loss, which was water loss, occurred between approximately 80–200 °C. In the case of the PB powder (O1), the next step (from 200 to 500 °C) corresponded to the exothermic decomposition of the cyano group of PB [37]. Additionally, above 500 °C a slight mass increase was observed, which was most likely caused by iron oxide formation. The pectin-only sorbent (S) started to decompose above 200 °C, which was seen as a rapid mass loss, and complete decomposition was observed at ca. 880 °C. The decomposition of the cross-linked pectin (S) was also observed in the thermograms of the hybrid sorbents (S1, S2, and S3). Based on these results and the TG profiles of the hybrid beads, the PB loading in the hybrid beads was calculated and equaled 3.6 ± 0.3%, 25.6 ± 0.3%, and 43.5 ± 2.4% for the S1, S2, and S3 hybrid sorbents, respectively. The calculated PB loading in the hybrid sorbents was in good agreement with the assumed ratio of PB and pectin, especially for the S2 and S3 sorbents. The calculated loading of PB powder in the S1 sorbent was lower than assumed, probably due to its worse homogeneity.

**Figure 3:**
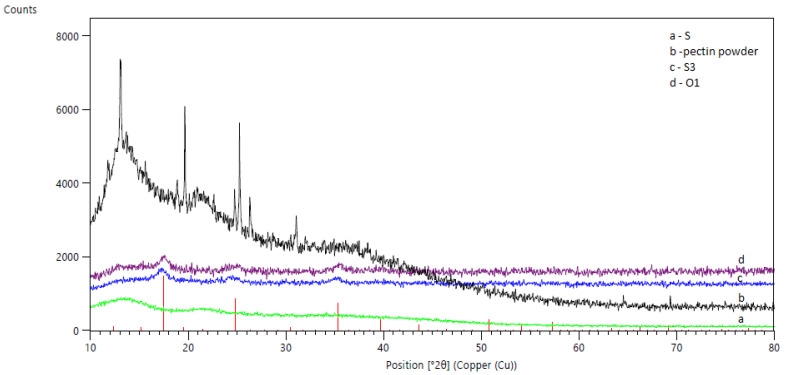
XRD patterns of: (**a**) S pectin-only sorbent, (**b**) pectin powder, (**c**) S3 hybrid sorbent, (**d**) O1 Prussian blue powder.

The crystalline properties of PB, pectin powder, and sorbents (hybrid and pectin-only) were examined by XRD analysis (Figure 3). The pectin-only powder (line b) showed four intense and distinct peaks (13°, 19°, 25°, and 31°, 2Theta), which indicated the crystalline nature of the pectin-only sample. The gelation process of pectin caused a loss of crystallinity, and the pectin-only sorbent showed an amorphous nature (line a). The X-ray diffractogram of the PB-only powder (O1, line d) had some characteristic peaks, but they were broad and not intense, which indicated the amorphous-crystalline nature of the synthesized powder. The hybrid sorbent S3, with the maximum PB content (line c), showed an amorphous nature, retaining characteristic peaks derived from the PB powder. 

**Figure 4:**
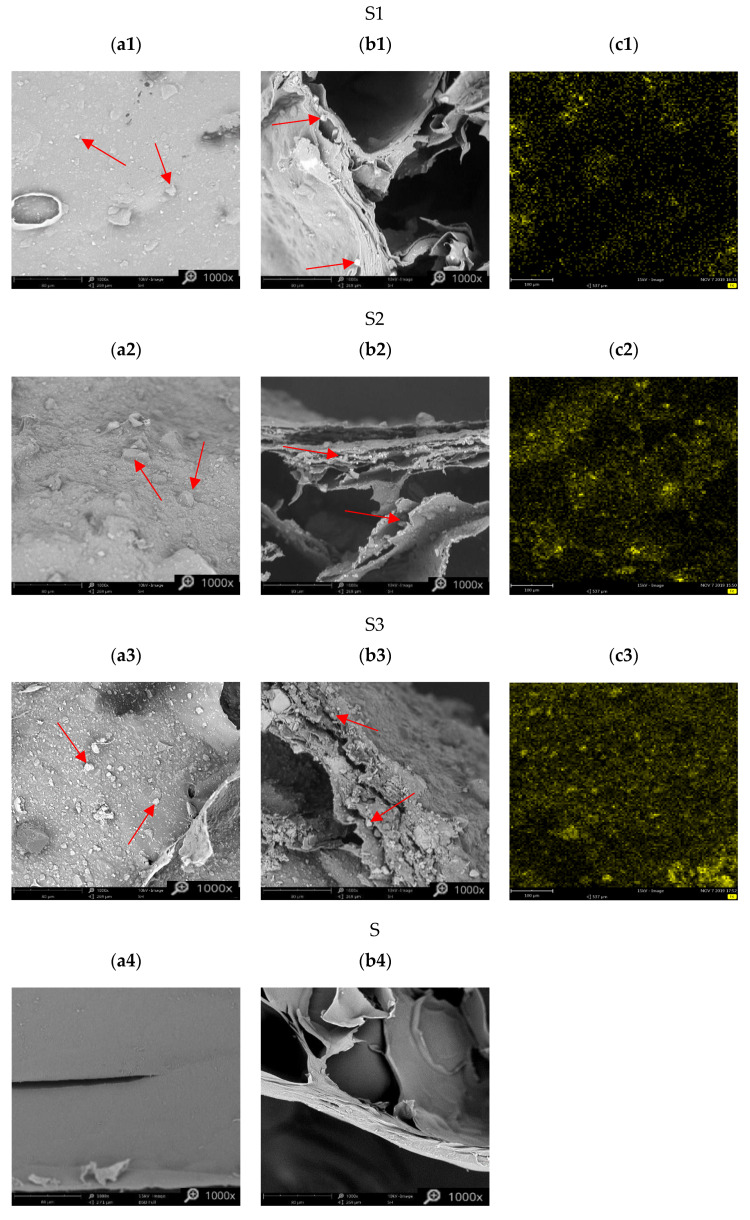
SEM micrographs of outer surface (**a1**–**a4**) (magnification 1000×), the cross-section (**b1**–**b4**) (magnification 1000×) and the Fe (yellow dots) EDS mapping (**c1**–**c3**) of hybrid sorbents with: 3.6% PB additive (S1), 25.6% PB additive (S2), 43.5% PB additive (S3), and the pectin-only sorbent (S).

In Figure 4, the micrographs of the outer surfaces (a) and the cross-sections (b) of the examined materials are presented. Both pectin-only and hybrid sorbents have a rather continuous outer surface with just few defects, but the cross section has a highly macroporous structure with the pore walls with a layered structure like a puff pastry. These layers seem to be less compressed for hybrid sorbents with PB powder (especially S3) than for pectin-only sorbent. The less dense structure of the hybrid sorbents is beneficial for the transportation of cesium ions into the active sites (PB powder) enclosed in the pectin matrix. The particles of PB are seen as lighter spots or small lumps embedded in the porous polymer in the S1, S2, and S3 hybrid sorbents (examples are marked with arrows). For sorbents with higher PB additive more of the PB powder can be seen in the SEM micrographs. The uniform distribution of PB into pectin was also confirmed by EDS analysis (Figure 4(c1–c3)). The yellow dots present the distribution of iron in the hybrid sorbents. The quantity of iron was greater for the sorbents containing higher PB additive (S2, S3). 

**Figure 5:**
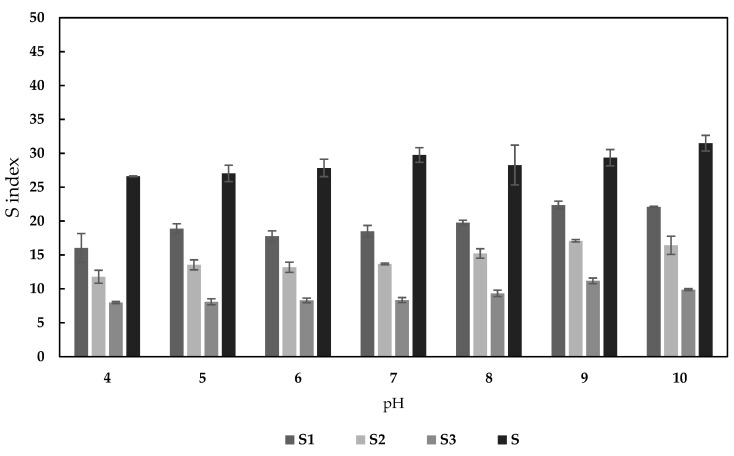
The effect of pH on the swelling index of sorbents (m = 30 mg, V = 10 mL, c = 115 mg/L, temp. = 22 ± 1 °C, t = 24 h).

The swelling of the sorbents during the sorption process was examined in a series of solutions with various pH values ranging from 4–10. The effect of pH on the swelling index of each sorbent is presented in Figure 5. The swelling ratio for all the sorbents increased slightly as the pH became more basic. The highest swelling index in the studied pH range was found for the pectin sorbent (31.5 ± 0.1). The increase in the PB content of the sorbent resulted in a decrease in its swelling index. For example, at pH = 6 the addition of PB powder reduced the S1, S2, and S3 sorbents’ swelling indices by 36%, 51%, and 70%, respectively.

The sorbents’ stability was determined by measuring the leaching of iron ions from the sorbent. The results proved the good stability of the hybrid sorbents in the 3–9 pH range. Iron leaching depended on the pH of the cesium ion solution (Table 2). The highest amount of iron was leached from sorbents at pH 2, which may be due to the partial decomposition of the sorbent component (PB) in the acidic medium. After six days at pH 2, the amount of leached iron increased by as much as two times. It is known from the literature that pectin sorbents give good stability only at a pH above 4 [26]. Our hybrid sorbents seemed to be stable even at pH 3.

PB undergoes partial decomposition in basic conditions (especially above pH 6.4) as a result of the reaction of iron ions with hydroxide ions [38,39]; in the case of our sorbents, however, this phenomenon was not observed. In alkaline conditions, the largest (but still small) amount of iron was washed from the S1 sorbent, which had the smallest amount of PB additive.

### 3.2. Preliminary Sorption Studies

**Figure 6:**
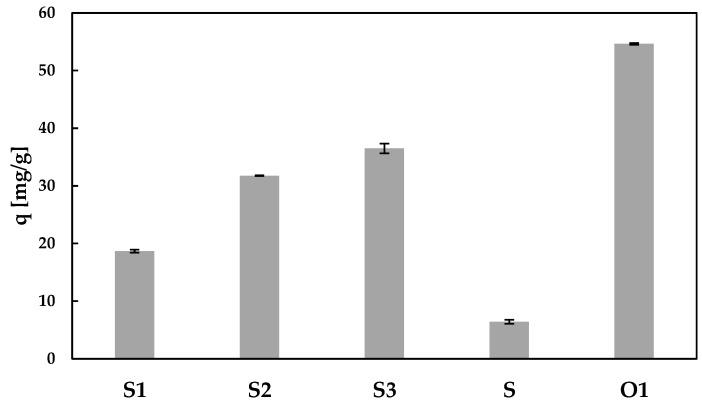
Sorption capacity of sorbents with 3.6% (S1), 25.6% (S2), and 43.5% (S3) of PB additive, PB-only powder (O1) and pectin-only sorbent (S) (m = 30 mg, c = 115 mg/L, V = 10 mL, pH = 6, temp. = 22 ± 1 °C, t = 24 h).

The sorption properties of prepared hybrid sorbents (S1, S2, S3) and pectin-only sorbent (S) were determined in a batch studies in the same conditions (mass of sorbent: m = 30 mg, volume of cesium ions solution: V = 10 mL, cesium ions concentration: c = 115 mg/L, pH = 6, temperature: 22 ± 1 °C and time: t = 24 h). Results are presented in Figure 6. In addition, the sorption capacity of sole PB powder (O1) has been placed (determined in the same conditions). The sorption property of the pectin-only sorbent was rather poor (its sorption capacity q = 6.4 ± 0.4 mg/g) and probably caused by the physical entrapment of cesium ions inside the sorbent beads and on their outer surface. Much better results were achieved using the hybrid sorbent, which indicates that PB is the main Cs sorption component. The sorption capacity of the materials increased with an increase in PB content, thus with an increase in the amount of the active substance with sorption centers. Sorbents with a 3.6%, 25.6% and 43.5% content of PB (S1, S2 and S3) had a sorption capacity of 18.7 mg/g, 31.8 ± 0.1 mg/g and 36.5 ± 0.8 mg/g, respectively (Figure 6). An evident synergistic effect was also noted in the hybrid sorbent. Taking into account that the sorption capacity of PB powder (O1) and sole pectin sorbent (S) equaled 54.6 and 6.4 mg/g, respectively, the sorption capacity of sorbent (S3) consisting of 43.5% PB and 56.5% pectin should be 27.4 mg/g (as weighted average), but was 36.5 mg/g. The same calculations for S1 and S2 sorbent of 3.6% and 25.6% PB content indicated that computed, based on particular component sorption capacities, theoretical values (8.1 and 18.7 mg/g, respectively) were much lower than experimental one (18.7 and 31.8 mg/g, respectively). The significantly higher than expected sorption may result from better PB dispersion in pectin matrix than in aqueous solution and thus from larger PB surface area in the hybrid sorbent. This effect may be similar to those achieved by Kadam et al. [29], who used a pectin as stabilizer preventing agglomeration of nanoparticles. Another explanation that does not exclude coexistence with the former may be that presence of PB powder changes the structure of pectin matrix and more cesium ions may be trapped into it; based on SEM images the structure of hybrid sorbents (S1, S2 and especially S3) seems to be less compact than pectin-only sorbent structure (Figure 4(b1–b4)). The difference between the determined sorption capacity and the predicted sorption capacity based on calculation increased with decreasing PB content in the hybrid material (sorption capacity increases of 131%, 70% and 33% for S1, S2 and S3, respectively). Sorption capacity calculated in relation to PB content also increased with decreasing PB content in the hybrid material (sorption capacity calculated in relation to PB content was of 519, 124 and 83.9 mg/g for S1, S2 and S3, respectively). An explanation may be that higher proportion of pectin matrix to PB in S1 sorbent ensures better PB powder dispersion and thus ensures greater increase in PB surface area. The important findings from these studies are that depending on the needs and possibilities (price of ingredients, technical conditions), the sorbent of the smallest total sorption capacity but the greatest sorption capacity calculated in relation to PB content (S1) or sorbent of the greatest total sorption capacity but the smallest sorption capacity calculated in relation to PB content (S3) may be obtained. It may be illustrated by the simplified example: removal of cesium from 1 liter of wastewater of Cs concentration 100 mg/l. In the first case (S1) more pectin (5.16 g) and greater amount of sorbent (5.35 g) (thus greater apparatus), but less PB (0.19 g) has to be used in comparison with the second case (S3), where less pectin (1.55 g) and smaller amount of sorbent (2.74 g) (thus smaller apparatus) but greater amount of PB (1.19 g) is needed to treat the same amount of cesium from wastewater.

Since this work is focused mainly on preparation and characterization of hybrid sorbents and studies concerning sorption are preliminary, the results cannot be the base of discussion on cesium sorption mechanism. However, it is worth mentioning, that it is generally hard to state what is the cesium ion sorption mechanism on the PB based sorbents. According to the literature, the sorption of cesium on PB powders even without any matrix is known to be a not fully explained process, composed of various independent phenomena: ion exchange, physical cesium ion entrapment, and physical sorption [20,40]. For composite materials, the sorption mechanism is more complex. In the case of our sorbents, the diffusion of cesium through the polymer matrix of pectin to the active ingredient (Prussian blue) may affects sorption and some cesium ions may be trapped into the crosslinked pectin matrix. The further extensive sorption studies are planned to investigate possible phenomena.

#### 3.2.1. Effect of the pH on Sorption Properties

**Figure 7:**
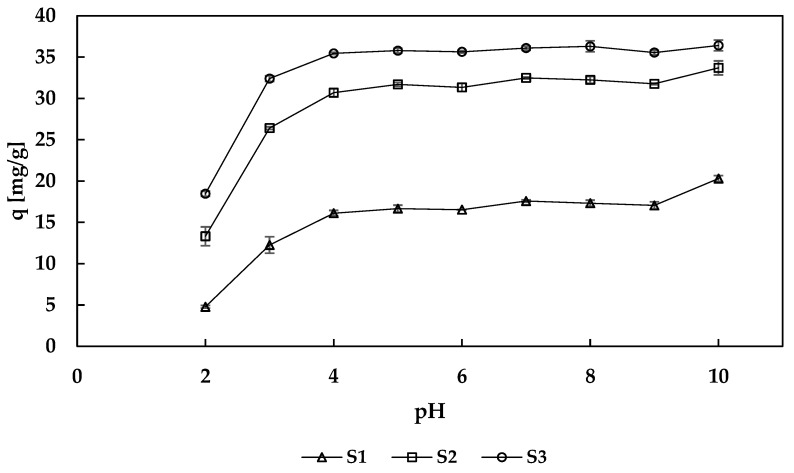
Effect of pH on sorption capacity (q) of sorbents (m = 30 mg, c = 115 mg/L, V = 10 mL, temp. = 22 ± 1 °C, t = 24 h).

The effect of pH on the sorption capacity of the hybrid sorbents was examined in the pH range 2–10 (Figure 7). The sorption capacity of pectin-PB beads reached a maximum at pH 4 and remained roughly constant over a wide pH range (4–10). This was also observed by some other authors for their PB-based materials [8,37]. At pH 3 amount of sorbed cesium ions decreased, but still was high. The lowest sorption capacities were noted when a solution of pH 2 was used. In such acidic solution the concentration of hydrogen ions is very high, and H^+^ ions may compete with the Cs^+^ ions for the active sites of the sorbent (this inhibits ion exchange—one of the phenomena occurring during the sorption of cesium on PB). Simultaneously, at low pH value PB may partially decomposed, thus some active sites may disappear and decrease the sorption capacity of the material.

#### 3.2.2. Effect of the Sorbent Dose on Sorption Capacity

**Figure 8:**
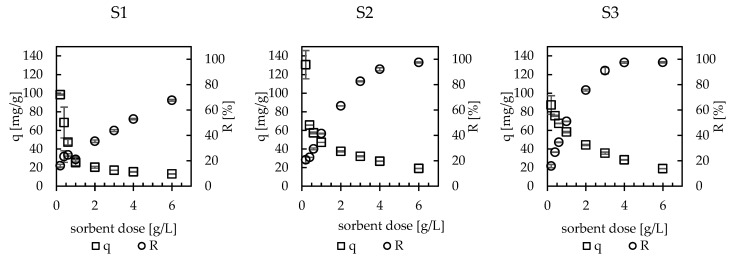
Effect of sorbent dose on sorption capacity (q) and removal efficiency (R) (c = 115 mg/L, V = 0.02 L, pH = 6, temp. = 22 ± 1 °C, t = 24 h).

The effect of sorbent dose on sorption capacity and cesium removal efficiency is presented in Figure 8. The sorption capacity expressed as the mass of cesium sorbed on 1 g of sorbent decreased with the sorbent dose increase, while the removal efficiency (expressed as % of cesium ions removed from the examined solution) increased and finally reached almost 100% for the S2 and S3 sorbents. For example, for the lowest applied dose of S2 hybrid sorbent (0.2 g/L), the sorption capacity was as high as 130.5 mg/g but the removal efficiency was low (21%). Under these conditions, the greatest amount of the active sites of the sorbent was occupied, and the excess of cesium ions was still in solution. For a sorbent dose of 6 g/L, the greatest amount of cesium ions was removed from the examined solution, with R = 98%, but the sorption capacity was very low (19.0 mg/g) (a lot of active sites on the sorbent were not occupied). 

Applying a sorbent dose of 3 g/L, the sorption capacities of the S1, S2, and S3 sorbents equaled 17.1 ± 0.1 mg/g, 32.1 ± 0.6 mg/g, and 35.6 ± 1.0 mg/g, respectively, and the removal efficiency was good (44%, 83%, and 91%). Therefore, a sorbent dose of 3 g/L, especially for the S2 and S3 sorbents, was found to be the best for cesium ion removal. These results proved, as mentioned above, that the sorption capacity strongly depends on the conditions of its determination, e.g., the sorbent dose, and this should be taken into account when the literature data for various sorbents are compared. 

## 4. Conclusions

New hybrid pectin sorbents with Prussian blue powder were obtained and preliminary tested for cesium ion removal. The preparation of Prussian blue powder (component) as well as the whole hybrid sorbents is easy to perform and automate. The obtained sorbents had a shape allowing them to be used in column systems and they were hard enough not to be crushed by finger rubbing. Another advantage of our round beads of sorbent is that they are easy to separate from the purified solution, as was proved during our batch studies. Additionally, the sorbents may be used over a wide range of pH (4–10), where the swelling and sorption properties are satisfactory. The sorbent beads also showed a good stability over this pH range, as proved by our examination of iron leaching. The studies proved that it is possible to remove even as much as 100% of the cesium ions using the obtained materials. The highest sorption capacity was achieved for the sorbent with the maximum Prussian blue additive (36.5 ± 0.8 mg/g). 

From a practical and technological point of view proposed method of PB hybrid sorbents preparation has some important advantages. All used precursors are readily available and cheap. Pectin is a natural waste product and is an interesting and cost-effective alternative for other proposed matrix substances (e.g., alginates, polyacrylonitrile). Cross-linking of pectin requires only non-toxic calcium salt, thus no difficult to manage sewage are produced. The process is fast and does not required use of complicated apparatus or extreme process conditions. This method can be successfully transferred to a larger scale. Additionally, synergic effect on sorption capacity for PB immobilized in pectin matrix was observed. An increase in pectin content increased the sorption capacity calculated in relation to PB content. It allows to adjust the composition of sorbent according to the needs and possibilities (price of ingredients, technical conditions). 

The conducted experiments showed that the obtained hybrid pectin beads are promising materials for cesium ion removal.

## Figures and Tables

**Table 1:** Parameters of Prussian blue (PB) powder synthesis and sorption capacity of PB-only powders.

Prussian Blue Powder	Ratio *	Conditions of Drying and Mixing	Sorption Capacity [mg/g]
O	1:1.15	-	-
O1	1:1.5	40 °C, 1 h	54.6 ± 0.1
O2	1:2.3	40 °C, 1 h	55.0 ± 0.4
O3	1:1.5	40 °C, 24 h	51.4 ± 1.0
O4	1:1.5	80 °C, 1 h	41.8 ± 1.6
O5	1:1.5	105 °C, 1 h	36.3 ± 0.4

* Molar ratio of K_4_[Fe(CN)_6_] to FeCl_3_.

**Table 2:** Hybrid sorbents’ stability-leaching of iron.

	% of Iron Leached from S1 Hybrid Sorbent (%)			% of Iron Leached from S2 Hybrid Sorbent (%)			% of Iron Leached from S3 Hybrid Sorbent (%)		
days	1	4	7	1	4	7	1	4	7
pH									
2	0.69	1.05	1.33	0.97	1.45	1.80	1.10	1.89	2.53
3	0.02	0.01	<0.01	0.01	0.01	0.01	0.01	<0.01	<0.01
4	0.04	0.03	0.03	<0.01	<0.01	<0.01	<0.01	<0.01	<0.01
5	0.06	0.04	0.03	0.02	<0.01	<0.01	<0.01	<0.01	<0.01
6	0.04	0.03	0.03	0.02	0.01	<0.01	<0.01	<0.01	<0.01
7	0.05	0.04	0.03	0.04	0.02	0.01	<0.01	<0.01	<0.01
8	0.04	0.03	0.03	0.03	0.01	0.01	0.01	<0.01	<0.01
9	0.07	0.06	0.09	0.02	<0.01	<0.01	<0.01	<0.01	<0.01
10	0.06	0.06	0.06	0.05	0.04	0.21	0.01	<0.01	<0.01
